# Emergence of ST8-USA300 and ST8-USA300-Latin American variant: a changing landscape of community-associated methicillin-resistant *Staphylococcus aureus* in Chile

**DOI:** 10.1128/spectrum.01031-25

**Published:** 2025-09-30

**Authors:** Alejandro Aguayo-Reyes, Felipe Morales-León, Mario Quezada-Aguiluz, Andrés Opazo-Capurro, Helia Bello-Toledo, Sergio Mella, Néstor Herrera-Chávez, Maximiliano Matus-Köhler, Juan Carlos Hormazábal, Gerardo González-Rocha

**Affiliations:** 1 Gupo de Estudio de Enfermedades Infecciosas (GrEEn-UdeC), Departamento de Medicina Interna, Facultad de Medicina, Universidad de Concepción, Concepción, Chile; 2 Grupo de Estudio en Resistencia Antimicrobiana (GRAM), Universidad de Concepción, Concepción, Chile; 3 Departamento de Farmacia, Facultad de Farmacia, Universidad de Concepción, Concepción, Chile; 4 Laboratorio de Investigación en Agentes Antibacterianos (LIAA), Departamento de Microbiología, Facultad de Ciencias Biológicas, Universidad de Concepción, Concepción, Chile; 5 Escuela de Tecnología Médica, Facultad de Medicina, Universidad Andrés Bello, Concepción, Chile; 6 Subdepartamento de Enfermedades Infecciosas, Instituto de Salud Pública de Chile, Santiago, Chile; Universidade de Sao Paulo, Sao Paulo, Brazil

**Keywords:** *Staphylococcus aureus*, CA-MRSA, USA300 clone, molecular epidemiology

## Abstract

*Staphylococcus aureus* is a versatile pathogen capable of causing various infections. Since the 1960s, the emergence of methicillin-resistant *S. aureus* (MRSA) has posed a significant challenge due to its antibiotic resistance. Among these, community-associated MRSA (CA-MRSA) emerged in the 1990s, which traditionally harbors the *mecA* gene and the Panton-Valentine leukocidin, affecting individuals without traditional risk factors. In this study, we aimed to describe the genetic and phenotypic characteristics of 54 CA-MRSA isolates circulating in Chile, collected between 2007 and 2023 from various healthcare centers across the country. All strains carried *mecA*, with SCC*mec* IVc being the most prevalent (67%). The predominant clone was USA300-Latin American variant (USA300-LV) (ST8-SCC*mec* IVc-COMER+) 43%, followed by USA300 (ST8-SCC*mec* IVa-ACME+) 15%, ST30-SCC*mec* IVc (15%), and ST5-SCC*mec* IVa (9%). All isolates were resistant to cefoxitin, and some exhibited additional resistance to erythromycin (18%), clindamycin (2%), and tetracycline (2%). Further studies on the molecular epidemiology of CA-MRSA in Chile should be performed, as our results evidenced the clonal nature and evolution of CA-MRSA in Chile, where the circulation of the ST8-USA300 and ST8-USA300-LV lineages in the community represents a serious threat for national public health.

**IMPORTANCE:**

The emergence of community-associated methicillin-resistant *Staphylococcus aureus* (CA-MRSA) poses a significant public health threat. Unlike hospital-associated strains, CA-MRSA affects healthy individuals outside healthcare settings, often resulting in severe infections. The World Health Organization has designated MRSA as a priority pathogen due to its association with life-threatening infections worldwide. The increasing prevalence of CA-MRSA strains, particularly those resistant to multiple antibiotics, further complicates treatment efforts. Our findings highlight the predominance of the ST8-USA300 and ST8-USA300-Latin American variant clones in Chile, indicating the ongoing evolution of CA-MRSA in the region and suggesting the potential establishment of these clones within the community. Monitoring the spread and genetic diversity of these strains is essential for developing targeted interventions and informing public health strategies to control this pathogen and mitigate its impact on national health.

## INTRODUCTION

*Staphylococcus aureus* is a major human pathogen responsible for a wide range of clinical manifestations, from skin and soft tissue infections to life-threatening conditions such as bloodstream infections, endocarditis, and pneumonia. Its adaptability and ability to develop resistance to multiple antibiotics, particularly methicillin-resistant *S. aureus* (MRSA), continue to pose serious public health challenges (1). MRSA remains a leading cause of healthcare and community-associated infections worldwide, associated with high mortality and morbidity rates (2). Recent global surveillance reports underscore the global ongoing burden of MRSA, emphasizing the need for effective prevention and treatment strategies (3).

For the first 30 years after its identification, MRSA infections were predominantly hospital-acquired, linked to healthcare-associated MRSA (HA-MRSA) strains (4). However, since the 1990s, community-associated MRSA (CA-MRSA) infections began to emerge (5). In addition, CA-MRSA has been associated more frequently with the expression of Panton-Valentine leukocidin (PVL) and a more antibiotic-susceptible phenotype (6, 7).

The dissemination of CA-MRSA is clonal and varies geographically, exhibiting dynamic changes over time. This complex phenomenon is influenced by multifactorial mechanisms that are not yet fully understood (8–10). In South America, recent studies have identified the predominant circulating genotypes as clonal complexes (CC) 30, 5, and 8. Specifically, these include ST30-SCC*mec* IVc (related to the Southwest Pacific clone), ST5-SCC*mec* IVa, and the Latin American variant of USA300 (USA300-LV) ST8-SCC*mec* IVc (11–13).

Early molecular epidemiology studies in Chile identified the Chilean/Cordobés clone (ST5-SCC*mec* I) as the main HA-MRSA lineage (12, 14). Until the mid-2000s, community-acquired infections involved mainly methicillin-susceptible *S. aureus*. In 2006, the first CA-MRSA cases emerged, linked to international travel and genetically related to the USA300 clone (15), prompting surveillance by the Chilean Institute of Public Health (ISPCh). Recent data from a tertiary care center in the Metropolitan Region show a decline in the Chilean/Cordobés clone prevalence, with the emergence of ST105-SCC*mec* II and ST72-SCC*mec* IV/VI (16). Additionally, increasing evidence supports the presence of ST8 CA-MRSA strains as causative agents of infection (16–18). These findings suggest a clonal replacement process in Chile, with rising diversity and the emergence of community-associated genotypes.

Despite the date since CA-MRSA was first reported, no large-scale studies have characterized Chilean isolates. Hence, this study aims to describe the genetic and phenotypic features of circulating CA-MRSA strains from the Chilean National Surveillance Program to better understand their local molecular epidemiology.

## MATERIALS AND METHODS

### CA-MRSA isolates

We analyzed 54 non-duplicate, randomly selected CA-MRSA strains harboring *lukS/F-PV* genes collected between 2007 and 2023 from 17 collaborating centers located in seven cities (Fig. S1), all participating in the Chilean National Surveillance Program coordinated by the ISPCh. Briefly, the inclusion criteria were as follows: *S. aureus* infections resistant to cefoxitin (FOX), diagnosed in patients from the community or within 48 hours of hospital admission, as long as the patient did not present any of the following healthcare-associated MRSA risk factors: hemodialysis, recent surgery, prolonged hospitalization, presence of a permanent catheter, or a history of previous MRSA isolation (19). It is important to note that, in practice, isolate referral to the ISPCh may also be based on the identification of suspected CA-MRSA strains through their antimicrobial resistance profiles—typically characterized by resistance to β-lactams and susceptibility to non-β-lactam agents (i.e., FOX-resistant and/or FOX-resistant plus erythromycin [ERY]-resistant)—and ultimately relies on the expert judgment of microbiology laboratory personnel. The strains were primarily isolated from skin and soft tissue specimens (*N* = 50/54) and bloodstream infections (*N* = 4/54). All isolates were cryopreserved at −80°C using a solution of glycerol (50% vol/vol) and trypticase broth (2:1).

### Antimicrobial susceptibility testing

Methicillin resistance was determined using the FOX disk (30 µg) diffusion test (20). Additionally, the antimicrobial susceptibility of the strains to ERY (15 µg), clindamycin (CLI, 2 µg), trimethoprim/sulfamethoxazole (SXT, 25 µg), rifampicin (RIF, 5 µg), and tetracycline (TET, 30 µg) was evaluated using the disk diffusion test, following the guidelines of the Clinical and Laboratory Standards Institute (CLSI) (20, 21). Furthermore, the minimum inhibitory concentrations (MICs) of vancomycin (VAN), linezolid (LZD), and daptomycin (DAP) were determined using the broth microdilution method according to the CLSI guidelines (20, 22).

### DNA extraction, sequencing, and genomic analyses

Genomic DNA of the CA-MRSA isolates was extracted using the Chelex 100 matrix (Bio-Rad, USA). Whole-genome sequencing (WGS) of the extracted DNA was performed using the Illumina NextSeq 2000 platform at SeqCenter (Pittsburgh, USA), generating 2 × 151 bp paired-end reads. The raw reads were assembled using SPAdes v.3.15.3. Quality control was performed by QUAST v.5.2.0 (# contigs <1,000) and CheckM v.1.2.3 (completeness >95% and contamination <5%). Further details regarding these strains are provided in the supplementary material (Table S1). The sequence types of the isolates were identified using the multilocus-sequence typing (MLST) tool hosted on the Galaxy Australia server (https://usegalaxy.org.au). For the detection of resistance and virulence genes, the National Center for Biotechnology Information (NCBI) Bacterial Antimicrobial Resistance Reference Gene Database and ResFinder database were used. Resistance genes were included in the analysis only if they exhibited a minimum identity and coverage of 90% for both. For virulence genes, the Virulence Factor Database was utilized with a threshold of 90% identity. All gene screening analyses were performed using ABRicate v.1.0.1. Subsequently, the SCC*mec* types and *spa* types of the isolates were determined using SCCmecFinder v.1.2 (https://cge.food.dtu.dk/services/SCCmecFinder/) and spaTyper v.1.04 (https://cge.food.dtu.dk/services/spaTyper/), respectively.

Additionally, genes related to copper and mercury resistance (COMER) and arginine catabolic mobile elements (ACME) were identified through BLAST analysis, as previously described (18).

A phylogenetic tree was constructed to elucidate the phylogenomic relationships of the CA-MRSA isolates. For this purpose, 30 CA-MRSA reference genomes from the NCBI database were selected as representatives of epidemic lineages or sequence types, including USA300, USA300-LV, ST5-SCC*mec* IVa, and ST30-SCC*mec* IVc from various countries in the Americas, and were originally characterized previously (11, 12). The core genome of the isolates was determined using Roary v.3.13.0, which identifies and aligns core genes shared across all genomes. Then, the phylogeny was inferred using the GTR+CAT model within a maximum-likelihood framework, using FastTree v.2.1.10 for rapid tree construction with the default parameters (23). The resulting tree was visualized and edited using the Interactive Tree of Life (iTOL) tool, allowing for detailed annotation (24). The final phylogenetic tree was used to explore the genetic diversity and relatedness of the CA-MRSA isolates in the study.

Additionally, to generate the single nucleotide polymorphism (SNP)-distance matrix, the alignment output file generated by Roary was analyzed using the SNP Distance Matrix V.0.8.2 tool available on the Galaxy Australia platform. Finally, the matrix values were plotted as a heatmap using Seaborn and MATLAB (Fig. S2).

## RESULTS

### Antimicrobial susceptibility testing

Resistance to FOX was confirmed in all strains analyzed (*N* = 54). Additionally, 10 strains (18.5%) exhibited resistance to ERY, with one strain also showing resistance to TET. All isolates remained susceptible to RIF, SXT, VAN, LZD, and DAP. The MIC_50_ values for VAN, DAP, and LZD were ≤0.125 µg/mL, 0.25 µg/mL, and 1 µg/mL, respectively, while the MIC_90_ values for VAN, DAP, and LZD were 0.125 µg/mL, 0.25 µg/mL, and 2 µg/mL, respectively.

### Genomic analyses

**Fig 1:**
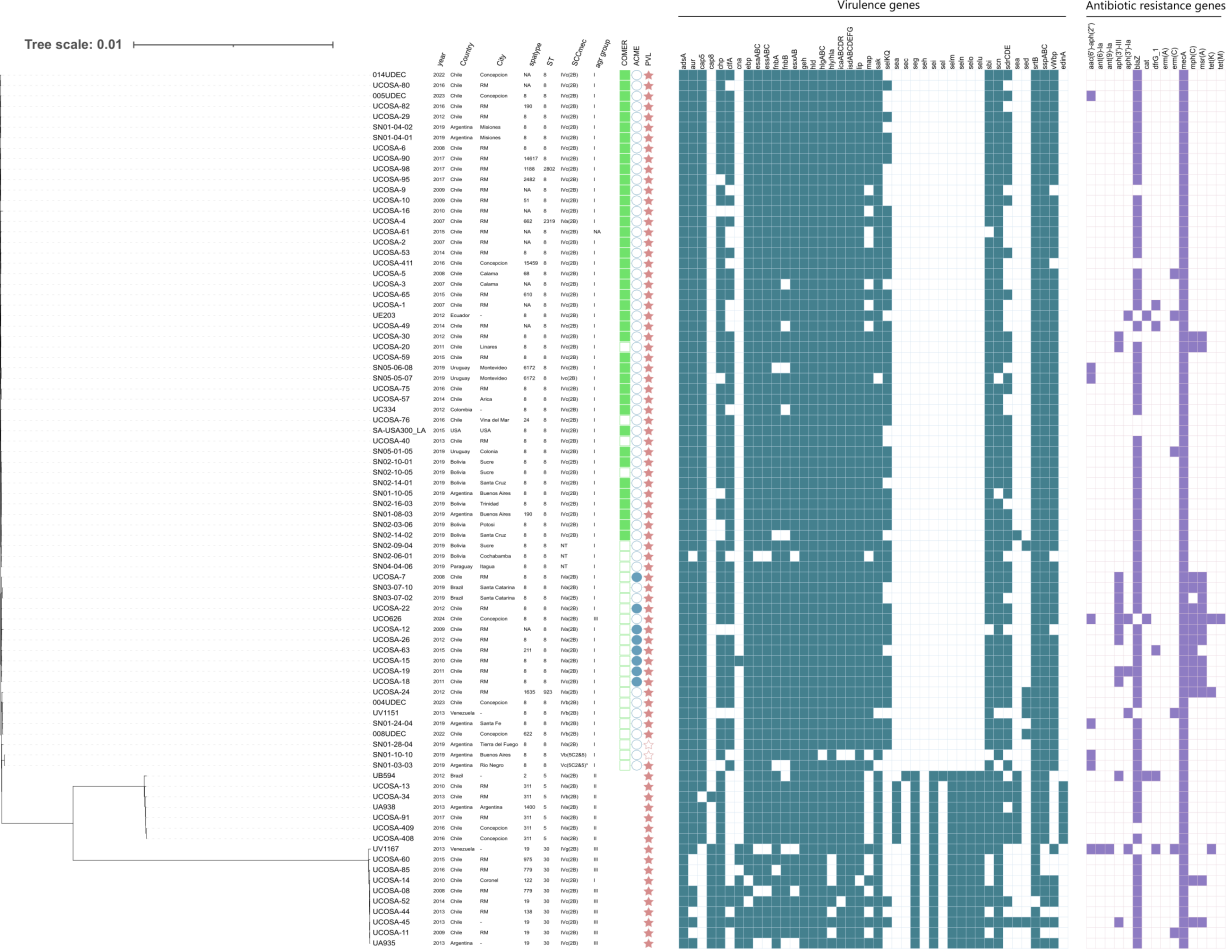
Core genome SNP phylogeny of CA-MRSA from Chile. Representative CA-MRSA genomes from isolates circulating in The Americas were included as references (see supplementary material). The phylogenetic tree was visualized using the online iTOL (v.6.0) and edited utilizing Inkscape software (version 1.2). ST, sequence type; SpaType, staphylococcal protein A type; SCC*mec*, staphylococcal cassette chromosome *mec*; NA, not assigned. As the COMER and ACME elements are related to the ST8 clone, they were not screened among the ST5 and ST30 isolates.

All strains analyzed carried the *mecA* gene, the primary determinant of methicillin resistance. Additionally, local isolates (*N* = 54) were found to carry SCC*mec* type IV, with SCC*mec* IVc (67%) being the most frequent subtype identified (Fig. 1). In terms of virulence profiles, all 54 isolates exhibited a wide array of virulence genes associated with adherence and biofilm formation (*icaABCDR* 98%*, clfA* 64%*, ebp* 96%*, map* 68%*, sdrCDE* 63%), immune evasion (*sbi* 87%*, scn* 100%*, cap5* 83%*, cap8* 17%*, chp* 94%, *fnbpA* 98%, *fnbpB* 96%, *cna* 15%), siderophore production (*isdABCDEFG* 100%*, srtb* 98%), exotoxin production (*esaABC* 98%*, essABC* 98%*, esxAB* 98%*, edinA* 9%*, hld* 100%*, hlgABC* 100%*, hly/hla* 91%*, lip* 98%*, sea1*%*, sed* 7%*, selKQ* 61%), and exoenzyme activity (*adsA* 100%*, aur* 90%*, geh* 100%*, sak* 100%*, sspABC* 93%*, vWbp* 78%) (Fig. 1). The presence of the *lukS/F-PV* genes encoding for PVL was confirmed in all 54 strains analyzed (Fig. 1).

Moreover, some strains harbored antimicrobial resistance genes to non-β-lactam antibiotics, including aminoglycosides (*aac(6')-aph(2"*) 4%*, aph(3')-III* 18%*, aph(3')-Ia* 4%), macrolide-lincosamide-streptogramine B antibiotics (*ermC* 4%*, mphC* 26%*, msrA* 24%), and tetracyclines (*tet(K*) 4%, *tet(M*) 2%), with one strain also harboring resistance to chloramphenicol (*cat*) 2%.

Most of the strains belonged to the ST8, with the predominant clone being USA300-LV (ST8-SCC*mec* IVc-COMER+) (*N* = 23/54), followed by USA300 (ST8-SCC*mec* IVa-ACME+) (*N* = 8/54) (Fig. 1). Additionally, three single-locus variants (SLVs) of ST8 were identified: ST2802, ST2319, and ST923 (Fig. 1). In seven of the isolates from CC8 (ST8 or its SLVs), neither the ACME nor the COMER elements were found. The third most prevalent clone corresponded to ST30-SCC*mec* IVc (*N* = 8/54), while the least represented was ST5-SCC*mec* IVa clone (*N* = 5/54) (Fig. 1). Interestingly, the USA300-LV clone was detected consistently from 2007 through 2023 among the isolates collection, indicating its long-standing and sustained presence in the community. In contrast, the USA300 clone appeared more sporadically, with a concentration of isolates in the earlier years of the study period (Fig. 1). Also, the USA300-LV clone was the most geographically widespread, identified in nearly all sampled cities, suggesting its successful dissemination across the country. Conversely, ST30-SCC*mec* IVc and ST5-SCC*mec* IVa clones were more geographically restricted, with most isolates originating from central regions, particularly the Metropolitan Region.

Among the 54 CA-MRSA isolates from Chile, *spa* typing revealed a total of 20 distinct *spa* types (Fig. 1). The most common was *t008*, predominantly associated with the USA300 and USA300-LV lineages. Other *spa* types, such as *t1635*, were linked to the ST923-SCC*mec* IVa lineage, while *t019* and *t779* were observed in isolates belonging to the ST30-SCC*mec* IVc clone. Furthermore, ST5-SCC*mec* IVa isolates were primarily associated with *spa* type *t311*.

## DISCUSSION

Our study provides a comprehensive overview of the molecular epidemiology of CA-MRSA in Chile, revealing the predominance of the USA300-LV (ST8-SCC*mec* IVc-COMER+) clone. In our study, all isolates carried *mecA* and SCC*mec* IV, consistent with CA-MRSA characteristics (7, 8). Notably, USA300 strains exhibited the broadest resistome (*aph(3’)-III*/*mphC*/*msrA*), particularly to ERY, reflecting their evolutionary success and blurring the line between CA- and HA-MRSA (7, 25, 26). In this context, the phenotypic detection of CA-MRSA, as currently performed in our country, may lead to an underestimation of the true epidemiological burden and genomic variability of these strains. Given the availability of advanced genomic tools such as WGS, we propose that in Chile, all MRSA isolates—both CA-MRSA and HA-MRSA—should be incorporated into a comprehensive surveillance framework. This approach is particularly important considering the increasingly indistinct boundary between these two MRSA populations.

The analysis of the virulome shows a clear distinction in capsular polysaccharide (*cap*) operons and accessory gene regulator (*agr*) types observed among the major CA-MRSA clones. Both USA300 and USA300-LV harbored the *cap5* operon and belonged to *agr* type I. In the ST5 SCC*mec* IVa clone, 80% of isolates carried *cap5* and 20% *cap8*, all classified as *agr* type II. In contrast, the ST30-SCC*mec* IVc lineage uniformly possessed *cap8* and *agr* type III. These molecular markers are established criteria for clonal assignment and typification (27, 28). Besides these markers, we identified a conserved virulence backbone across lineages—genes such as *adsA, aur, chp, esaABC, essABC, esxAB, fnbA, hld, hlgABC, hla, icaABCDR, isdABCDEFG, lip, sak, sbi, scn, sdrCDE, sspABC, and srtB*—present in nearly all isolates. This common set supports the basal pathogenic potential of CA-MRSA in Chile.

However, our data also reveal significant variability. The ST8 lineage (USA300 and USA300-LV) exhibited a differential virulome compared to the CC5/ST5 and CC30/ST30 (Fig. 1), in which these latter harbored the enterotoxin gene cluster (EGC) that is not present in the CC8 (Fig. 1). Moreover, ST8 strains consistently displayed adhesins and immune evasion factors—*clfA, fnbB, map, selKQ, and vWbp*—which reinforce their hypervirulent and biofilm-forming capabilities.

In contrast, ST5-SCC*mec* IVa lacked *map, selKQ, and vWbp*, but uniquely harbored the *edinA* gene—previously reported in Paraguay—alongside the EGC (29). The latter was also detected in ST30 SCC*mec* IVc isolates, suggesting shared toxin-mediated virulence strategies. The presence of EGC toxins, which are common in clinical isolates, can exacerbate infections like respiratory diseases and endocarditis by intensifying immune responses, potentially leading to severe complications such as heart failure and stroke (30). Interestingly, ST30 showed slightly lower prevalence of *icaABCDR, lip, and srtB* (88%), yet maintained key adhesins like *cna, fnbA, and fnbB,* supporting its persistence in community-associated infections.

These findings align with Guillén et al., who reported diverse virulence and resistance profiles in CC5, CC8, and CC30 lineages in Paraguay (29). Our results support that while CA-MRSA clones share a core virulence profile, lineage-specific elements like COMER, ACME, EGC, and *edinA* contribute to differences in adaptation and pathogenicity. We also identified siderophore-related genes (*isdABCDEFG* and *srtB*), which regulate iron homeostasis and enhance replication and infection severity. The *Isd* system also aids in adhesion and resistance to neutrophils (31). Multiple genes related to adherence, immune evasion, exotoxins, and exoenzymes were present, reinforcing the role of virulence factors in MRSA severity (32). Moreover, all analyzed strains carried genes encoding the PVL toxin (*lukS/F-PV*). Although the presence of this leukocidin has been epidemiologically associated with CA-MRSA since its emergence, not all CA-MRSA strains harbor the *lukS/F-PV* genes. Thus, PVL detection should not be considered the defining or exclusive marker of community-associated isolates (7, 33).

Epidemiologically, our results revealed the presence of four predominant CA-MRSA clones: (i) USA300-LV (ST8-SCC*mec* IVc-COMER+); (ii) USA300 (ST8-SCC*mec* IVa-ACME+); (iii) ST30-SCC*mec* IVc; and (iv) ST5-SCC*mec* IVa. In this sense, these clones are predominant globally, as well as in South America, and were also previously confirmed as the main CA-MRSA lineages in the region in a prospective observational study conducted in 2019, which included 58 hospitals from Argentina, Bolivia, Brazil, Paraguay, and Uruguay (11). Furthermore, the observed associations between *spa* types, SCC*mec* elements, and STs support the clonal structure of CA-MRSA in Chile. For instance, the predominance of *spa* type *t008* among ST8 isolates is consistent with its global association with the USA300 lineage. Locally, the detection of *spa* type *t1635* in ST923 isolates aligns with reports from Colombia, where this clone has been described as genetically distinct from USA300 despite being an SLV of ST8 (34). Additionally, the analysis of the strains from this study, alongside representative strains isolated from other South American countries (Fig. 1 and Fig. S2), revealed that similar STs clustered together regardless of their country of origin. At this point, it is important to highlight that, although the emergence of CA-MRSA in the region has been variable—affecting some countries earlier than others—the epidemic is generally being driven by the same successful clones, with some country-specific variations. For example, ST8-SCC*mec* IV predominates in Bolivia, ST30-SCC*mec* IV in Argentina and Paraguay, whereas ST5-SCC*mec* IV has been also importantly described in Argentina and Brazil. Although all three clones were documented in Chile, there has been a clear predominance of ST8-SCC*mec* IV. The results are consistent with previous reports of MRSA isolates collected in South America (11, 12, 29), reinforcing the clonal nature of MRSA spread in the region. Collectively, these observations underscore the importance of sustained molecular surveillance to monitor the dynamics and expansion of dominant MRSA clones across South America.

In the specific case of the USA300-LV (ST8-SCC*mec* IVc-COMER+) clone, this was originally described in 2005 and has since become a major cause of CA-MRSA infections across the continent, particularly in the northern countries of South America (35). In this sense, our findings indicate that USA300-LV was the most prevalent and widely distributed CA-MRSA lineage in Chile between 2007 and 2023. This clone was detected across multiple regions and years, suggesting its sustained presence in the community. As mentioned above, this clone was restricted to countries located in the northern region of South America, including Colombia, Ecuador, and Venezuela (12). Remarkably, recent genomic data indicate its broader dissemination across the region with heterogeneous prevalence. Specifically, the StaphNET-SA study identified this clone as the predominant MRSA lineage in Bolivia, where phylogenetic analysis suggests a single introduction event followed by local clonal expansion. In Argentina, Paraguay, and Uruguay, USA300-LV has been detected at lower frequencies, indicating sporadic transmission beyond its northern epicenter. In the case of Brazil, CC8 has been described at lower rates in comparison to other CCs, with CC5 being the predominant; however, there is evidence of more diverse CC8 sublineages, such as Iberian/CC8b, USA500/CC8c, and USA300 (11). In the local context, multiple factors may affect the prevalence and success of USA300-LV clone in the country, such as the presence of antibiotic or heavy metal resistance determinants, virulence-associated genes, and other related elements (10). Moreover, globalization and migration dynamics can contribute to the dissemination of these pathogens. In recent years, Chile has seen a substantial influx of immigrants from regions with a high prevalence of USA300-LV, raising the possibility that the burden of this pathogen within the country may escalate in the future (18). Interestingly, one of the strains included in this study belongs to the ST923-SCC*mec* IVa-*spa* type *t1635* lineage, previously detected in multiple regions of Colombia (clone COL923) (34). This SLV is particularly notable for its resistance genes against non-β-lactam antibiotics, including the *mphC* and *msrA* genes, which mediate resistance to macrolides, as well as the *tet*(*K*) gene, which provides resistance to tetracyclines (36). Additionally, our study revealed that one of the isolates is classified as ST2802, an SLV of ST8, which displays a similar resistance phenotype with the USA300-LV clone. While reports of this lineage are scarce, it has been previously documented in Chile in an isolate collected in a hospital in Santiago (16). Although resistance to DAP has been reported in an ST2802 MRSA isolate from Colombia (37), the strain analyzed in our study was susceptible to this drug.

In our study, we identified CA-MRSA isolates belonging to the ST30-SCC*mec* IVc lineage, also known as the Southwest Pacific clone. This lineage has been circulating in South America since the early stages of the CA-MRSA epidemic (12, 38–40) and has also been reported outside the region (13). Genomic studies have confirmed its persistence in countries such as Argentina and Paraguay, where ST30-SCC*mec* IVc (mainly *spa* type *t019*) became the predominant CA-MRSA clone in both community and healthcare settings (29, 41). However, recent surveillance data indicate a decline in its prevalence in some areas, such as Bolivia and Colombia, where emerging clones like USA300-LV are gaining ground (11). In our collection, eight ST30-SCC*mec* IVc isolates were identified, displaying notable *spa* type diversity. This contrasts with the dominance of *t019* reported in neighboring countries and may reflect local diversification or multiple introduction events of the clone into Chile. Notably, despite its historical prevalence, the ST30-SCC*mec* IVc clone was not detected in our collection after 2016 (Fig. 1), suggesting a replacement by other emerging CA-MRSA lineages in Chile, mirroring trends observed elsewhere on the continent.

Regarding the ST5-SCC*mec* IV clone, we identified five isolates belonging to this lineage, which exhibited genetic relatedness to contextual strains previously isolated in Brazil and Argentina, countries where this CA-MRSA clone was historically prevalent, particularly during the first decade of the 21st century (42, 43). This clone was historically a major CA-MRSA clone in South America, often associated with *spa* type *t311* and carriage of *lukS/F-PV* and *sea* genes, contributing to skin and soft tissue infections and invasive disease (29, 44). Consistent with these findings, our isolates also harbored both *lukS/F-PV* and *sea* and predominantly exhibited *spa* type *t311*. However, the prevalence of ST5-SCC*mec* IV has declined regionally, being replaced by CC5 basal clades with other *spa* types such as *t002* and *t509*, reflecting ongoing clonal diversification and possible SCC*mec* acquisition or loss within this lineage (11). As observed with the Southwest Pacific clone, these isolates were detected only during the initial period covered by our study, suggesting a possible clonal replacement over time, a pattern also reported in other countries across the region.

A key limitation of our study is originated from the structure of the Chilean Surveillance Program for CA-MRSA, which relies mainly on antibiotic susceptibility profiles—i.e., FOX-resistant and/or FOX-resistant plus ERY-resistant—to identify suspected community-associated clones. This approach may hinder the detection of community-associated clones that do not conform to this criterion. Additionally, our study primarily included *lukS/F-PV*-positive strains isolated from skin and soft tissue infections, limiting our ability to determine whether these isolates were colonizing or actively causing disease. A further limitation pertains to the geographic scope of the sampling, which encompassed strains from only 6 of Chile’s 16 administrative regions (Fig. S1), with a disproportionately large representation from the Metropolitan Region. Collectively, these limitations restrict the generalizability of our findings and may obscure regional variations in the molecular epidemiology of CA-MRSA. Future studies addressing these gaps could provide a more comprehensive understanding of the diversity and distribution of circulating clones across the country.

In conclusion, this study identified the circulation of four CA-MRSA clones in Chile—USA300-LV, USA300, ST30-SCC*mec* IV, and ST5-SCC*mec* IV—all of which have been previously reported in other South American countries. In this context, our results show the predominance of ST8 clones, mainly USA300-LV in the community, underscoring the dynamic and evolving nature of CA-MRSA epidemiology and emphasizing the urgent need for sustained comprehensive molecular surveillance. Moreover, this is particularly relevant for local CA-MRSA surveillance in both community and hospital settings, as has been observed in other geographical regions, where the boundaries between these two environments have increasingly become blurred. Such efforts are essential for accurately monitoring the emergence and replacement of circulating CA-MRSA clones and for guiding effective treatment strategies based on their molecular epidemiology.

## Data Availability

The genomes sequenced for this study are deposited in at GenBank under the BioProject number PRJNA1198102.

## SUPPLEMENTAL MATERIAL

Legend for Fig. S1 spectrum.01031-25-s0001.docx Description of Fig. S1.

Legend for Fig. S2 spectrum.01031-25-s0002.docx Description of Fig. S2.

Fig. S1 spectrum.01031-25-s0003.tiff Map depicting the cities included in this study.

Fig. S2 spectrum.01031-25-s0004.tiff Pairwise SNP matrix of the genomes analyzed.

Table S1 spectrum.01031-25-s0005.xlsx Detailed information of the strains included in this study.

ASM does not own the copyrights to Supplemental Material that may be linked to, or accessed through, an article. The authors have granted ASM a non-exclusive, world-wide license to publish the Supplemental Material files. Please contact the corresponding author directly for reuse.
