# Supplementary material for: Emergence of ST8-USA300 and ST8-USA300-Latin American variant: a changing landscape of community-associated methicillin-resistant Staphylococcus aureus in Chile
Source: Microbiol Spectr. 2025 Sep 30;13(11):e01031-25. doi: 10.1128/spectrum.01031-25 (PMC12584766; doi:10.1128/spectrum.01031-25)
Supplement: Legend for Fig. S2 — Description of Fig. S2. [file spectrum.01031-25-s0002.docx]

**Figure S2.** Pairwise SNP matrix of the 84 CA-MRSA included in this study.
