# Supplementary figures and images for: Emergence of ST8-USA300 and ST8-USA300-Latin American variant: a changing landscape of community-associated methicillin-resistant Staphylococcus aureus in Chile
Source: Microbiol Spectr. 2025 Sep 30;13(11):e01031-25. doi: 10.1128/spectrum.01031-25 (PMC12584766; doi:10.1128/spectrum.01031-25)

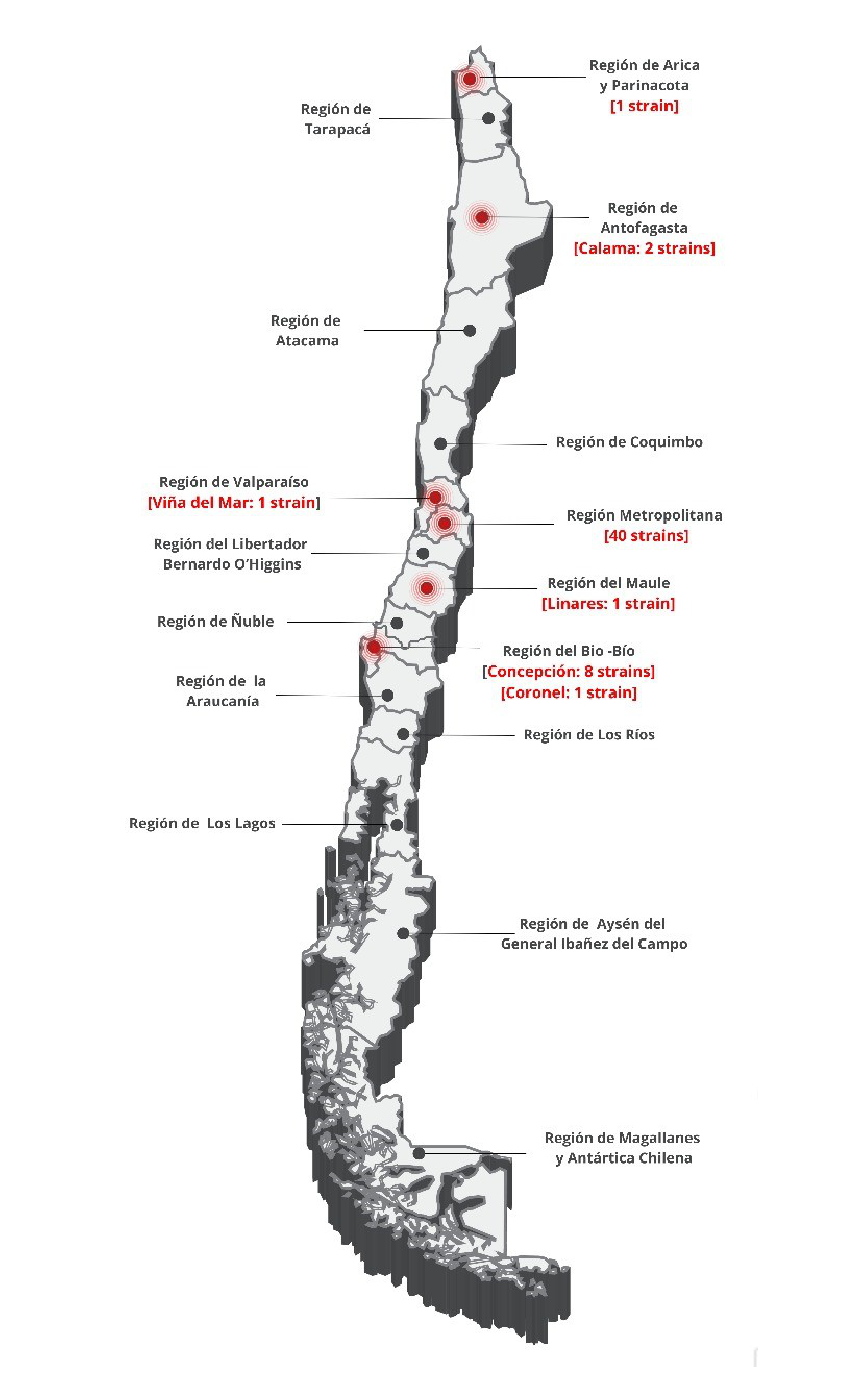

Supplement: Fig. S1 — Map depicting the cities included in this study. [file spectrum.01031-25-s0003.tiff]

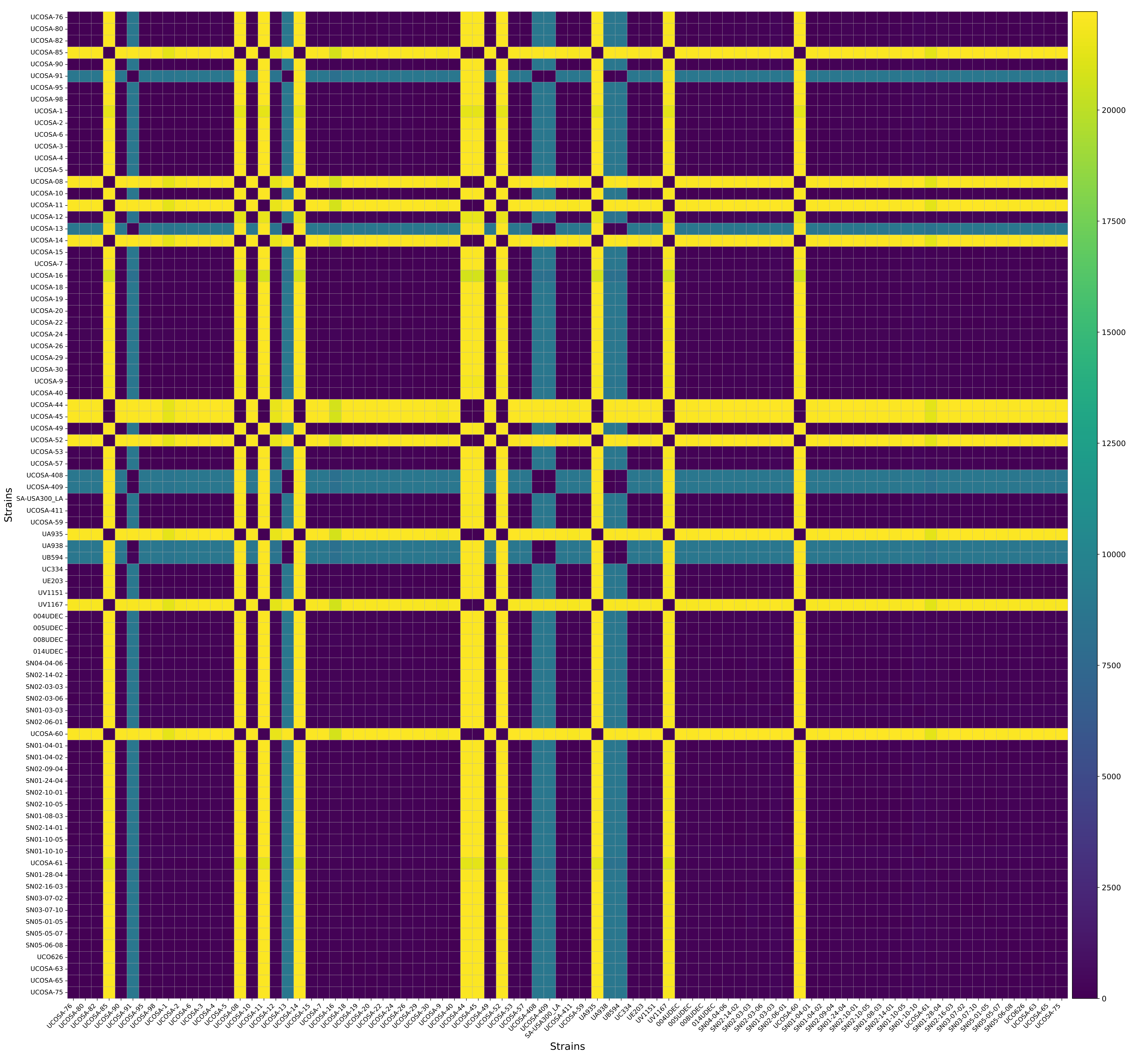

Supplement: Fig. S2 — Pairwise SNP matrix of the genomes analyzed. [file spectrum.01031-25-s0004.tiff]
